# Spatial epidemiology of tuberculosis diagnostic delays, healthcare access disparities, and socioeconomic inequities in Nairobi County, Kenya

**DOI:** 10.1371/journal.pone.0329984

**Published:** 2025-08-08

**Authors:** David Majuch Kunjok, John Gachohi Mwangi, Salome Kairu-Wanyoike, Johnson Kinyua, Susan Mambo

**Affiliations:** 1 Department of Environmental Health and Disease Control, School of Public Health, Jomo Kenyatta University of Agriculture and Technology, Juja, Kenya; 2 World Health Organization, Juba, South Sudan; 3 School of Biomedical, Jomo Kenyatta University of Agriculture and Technology, Juja, Kenya; Independent Consultant, UNITED STATES OF AMERICA

## Abstract

**Introduction:**

Kenya ranks among the top 30 countries with a high tuberculosis (TB) burden globally. With a TB prevalence of 558 per 100,000, only 46% of TB cases are diagnosed and treated, leaving 54% undiagnosed and at risk of spreading the disease. This study analyzed the spatial distribution of tuberculosis diagnostic delays and their association with health care accessibility and socioeconomic inequalities in Nairobi County, Kenya.

**Materials and methods:**

The cross-sectional study included 222 newly diagnosed bacteriologically confirmed *Mycobacterium tuberculosis* (Mtb) patients from Mbagathi County Hospital (MCH), Mama Lucy Kibaki Hospital (MLKH), and Rhodes Chest Clinic (RCC) in Nairobi County, Kenya. Patients were recruited consecutively through census sampling and categorized into two groups: delayed diagnosis (≥21 days from symptom onset) and non-delayed (<21 days) as defined by the WHO cutoff point. Patients’ residential locations were georeferenced using handheld GPS devices and captured digitally via Kobo Collect. Spatial analyses were performed using ArcGIS Pro, version, where Global Moran’s I statistic was used to assess spatial autocorrelation in the distribution of TB cases.

**Result:**

Spatial analyses identified 28 statistically significant clusters of delayed TB diagnoses within Nairobi County. Spatial autocorrelation analysis using Moran’s I revealed a significant clustered distribution (Moran’s Index = 0.471, z-score = 3.370, p < 0.001). Hotspot analysis with the Getis-Ord Gi* statistic detected high-delay clusters (z > 2.58, p < 0.001) in informal settlements.

**Discussion and conclusion:**

The study revealed significant spatial clustering of delayed TB diagnoses in Nairobi County, particularly in informal settlements. In contrast, timely diagnoses were predominantly clustered in high-income areas like Lang’ata and Karen. These clusters were significantly associated with lower household income and increased travel time to health facilities which underscored the need for targeted implementation of TB diagnostic services and control measures in the wards with the highest delays.

## Introduction

Tuberculosis (TB) remains the world’s leading cause of death from a single infectious agent, accounting for an estimated 1.25 million deaths globally in 2024 [1]. Although the number of newly diagnosed TB cases rose to 8.2 million in 2023, up from 7.5 million in 2022 and 7.1 million in 2019, diagnostic gaps persist in many high-burden countries. Globally, 87% of these cases are concentrated in 30 high TB-burden countries, including Kenya [1]. Delayed diagnosis contributes significantly to TB mortality, morbidity, and ongoing transmission, while also imposing substantial economic burdens on patients and healthcare systems [2]. The majority of people infected through community transmission may not develop the disease. However, those who develop the disease between infection and the development of signs and symptoms take weeks or decades [2].

In Kenya, TB is a major public health challenge, ranking fifth among the country’s top ten causes of death, with 4.7% (5,845), translating to 11 deaths per 100,000 population and among the world’s top 30 countries with a high TB burden. The estimated prevalence of bacteriologically confirmed pulmonary TB is 558 per 100,000 population in people 15 years and older [3]. TB ranks fifth in Kenya’s top ten causes of death [3]. Among the people who contracted TB (169,000) in 2016, only 46% (77,376) were diagnosed and administered recommended treatment, while the remaining 54% remained undiagnosed. The TB burden is highest in informal urban settlements, with Nairobi, Nyanza, and the coast regions leading [4]. Nairobi County contributes 15% of the cases of the ten leading counties [3].

The undiagnosed pulmonary TB acts as a reservoir for ongoing transmission, worsening disease severity, and leading to complications and high fatality rates. In addition, it increases TB transmission, resulting in 10–15 secondary TB infections annually, further straining the healthcare systems and increasing the cost and complexity of the treatment for affected individuals [5]. Existing TB surveillance systems are largely passive and rely on aggregate facility-level reporting, limiting the ability to identify geographic and sociodemographic gaps in access to diagnosis [6]. There is limited literature using geocoded, individual-level data to study spatial patterns of TB diagnostic delays. Understanding where delays occur and how they relate to socioeconomic and geographic factors is essential for targeted interventions. This study aims to address gaps by investigating the spatial distribution of delayed and non-delayed TB cases in Nairobi County, identifying high-risk areas, specifically, it sought to (1) identify clusters of delayed diagnosis, (2) assess the association between patient-level characteristics and diagnostic delay, and (3) explore healthcare access factors associated with delay patterns in Nairobi County, Kenya.

## Materials and methods

### Study site

Nairobi County is situated in the South-Central region of Kenya. The study was conducted in three public health facilities: Mbagathi County Hospital (MCH), Mama Lucy Kibaki Hospital (MLKH), and Rhodes Chest Clinic (RCC).. These facilities were randomly selected from a list of public health institutions providing tuberculosis (TB) services nationwide, ensuring geographic and demographic representation across different urban settings within Nairobi.

### Study design

A cross-sectional study was conducted between January and June 2024 among 222 newly diagnosed pulmonary TB patients aged 15 years and older.

### Case definitions

In this study, a tuberculosis (TB) case was defined as a newly diagnosed individual without a history of TB treatment who had not received anti-TB medication for more than one month, according to WHO guidelines.

Delayed diagnosis was characterized by an interval exceeding 21 days between the onset of typical TB symptoms and bacteriological confirmation via smear microscopy, culture, or GeneXpert testing. TB symptoms considered included persistent cough lasting more than two weeks (possibly blood-stained), fatigue, chest pain, fever, weight loss, night sweats, and general body weakness. A non-delayed diagnosis refers to bacteriological confirmation of TB within 21 days of symptom onset. Patient delay was defined as the duration from the onset of TB symptoms to the first presentation to healthcare facilities. Healthcare system delay refers to the time between the patient’s initial contact with a healthcare provider and the confirmation of a TB diagnosis [7].

### Target population

This study focused on smear-positive pulmonary tuberculosis (PTB) patients aged 15 and above within Nairobi County. Participants with no history of TB treatment and who initiated anti-tuberculosis therapy within one month preceding the enrollment were included in the study to ensure culture viability of *Mycobacterium tuberculosis*. Patients whowere referred from private or informal providers but received diagnostic confirmation and treatment initiation at the study sites were included in the study. Written informed consent was obtained from all participants before their inclusion in the study. Bedridden patients, those with a previous history of TB treatment, including relapse cases, participants with incomplete clinical records, and those with significant comorbidities that could confound diagnostic outcomes were excluded.

### Sample size population

The sample size was calculated using Cochran’s (1977) formula for estimating proportions in a finite population, assuming a 15% prevalence in Nairobi County, and the final sample size was 222 participants. This sample was proportionally distributed across the study facilities based on the number of smear-positive TB cases reported in 2022: 113 participants (50.9%) were drawn from Mbagathi County Hospital, 77 (34.7%) from Mama Lucy Kibaki Hospital, and 32 (14.4%) from Ngaira Rhodes Health Centre. This approach ensured that the sample reflected the TB burden distribution across the selected facilities.

### Sampling procedure

A census sampling technique was employed. Eligible smear-positive, newly diagnosed TB patients aged 15 years and above attended the selected health facilities from January to June 2024 and were recruited consecutively until the required sample size was achieved. Recruitment was conducted during scheduled clinic visits, and only patients who met the inclusion criteria and provided written informed consent were enrolled in the study. This method ensured comprehensive coverage of the target population during the study period and minimized selection bias.

### Data collection

Data was collected through an interviewer-administered survey using a semi-structured mobile-based Kobo Collect questionnaire. The questionnaire was divided into sections covering sociodemographic characteristics, including household income, which were self-reported by participants at the time of enrollment. Respondents estimated their average monthly household income in Kenya shillings and health-seeking behavior, which were obtained through interviews conducted between January and June 2024. Clinical data were collected from sputum samples during the same period, while the date of diagnosis was extracted from medical records covering the same period. Community Health Volunteers (CHVs) received an Informed Consent training that included Digital Data Capture and Structured Interviewing Skills. The internal consistency was excellent to acceptable, with reliability coefficients of 1.00 for items measuring delayed and non-delayed diagnosis and 0.700 for spatial data variables.

### Data analysis

This study employed an exploratory spatial analysis framework using individual-level geocoded data to assess the spatial distribution of diagnostic delays in tuberculosis care across Nairobi County. GPS coordinates were reviewed for accuracy using ArcGIS by overlaying patient locations on high-resolution base maps. Misaligned points were corrected by snapping them to the nearest relevant geographic feature (e.g., building, road, or health facility), and coordinates were standardized to WGS 1984. Duplicates were excluded from analysis.

Spatial analysis was conducted using geocoded patient-level data to assess patterns in TB diagnostic delays. Global Moran’s I evaluated overall spatial autocorrelation, while Getis-Ord Gi* identified significant hotspots and coldspots of delay. Anselin’s Local Moran’s I detected local clusters and spatial outliers by classifying areas into high-high, low-low, and outlier categories. Additionally, the Density-Based Spatial Clustering of Applications with Noise (DBSCAN) algorithm was used to visualize geographic concentrations of delayed and non-delayed cases. This integrated approach enabled both statistical and operational interpretation of spatial patterns in diagnostic access. Geocoded patient residence coordinates and the spatial distribution of time lags in TB diagnoses were analyzed with ArcGIS Pro version 10.8.2, which allowed for the identification of localized clusters of delayed diagnosis at the ward level, offering a level of spatial granularity. The WGS 1984 coordinate system was selected as the reference system for geospatial data accuracy and consistency. To assess whether delayed TB diagnoses demonstrated spatial clustering, dispersion, or randomness, global spatial autocorrelation was measured using Moran’s I. Moran’s I statistics ranging from −1 to +1 were considered significant; Values of +1 indicated spatial clustering, values near −1 suggested dispersion, and values of 0 implied a random distribution. Z-score with values ≥1.96 at p ≤ 0.05 denoting significant spatial autocorrelation. Further analysis was conducted to examine local spatial clustering by utilizing Local Indicators of Spatial Autocorrelation (LISA), in addition to cluster and outlier Analysis using Anselin Local Moran’s I for statistically significant clusters of high or low values (hot and cold spots) and spatial outliers.

The Getis-Ord Gi* statistic was used to analyze hot spots, identifying statistically significant spatial clusters of delayed TB diagnoses. This method detected areas with a spatial concentration of high values (hot spots) and low values (cold spots). A high positive Gi* Z-score indicated clustering of high values, while a low negative Z-score reflected clustering of low values. Statistical significance was assessed using Z-scores and *p*-values, with clusters considered significant at *p* ≤ 0.05 and Z ≥ 1.96.

Spatial accessibility to TB diagnostic services was analyzed through network-based analyses in ArcGIS, including Closest Facility, Service Area, and Location-Allocation models. These methods quantified travel distances, delineated catchment zones, and identified optimal facility locations to improve geographic coverage and reduce patient travel burden. All spatial data were standardized, validated, and analyzed using consistent geoprocessing protocols, visualizing outputs to inform equitable health service distribution and planning.

### Ethical considerations

This study received ethical approval from the Kenyatta University Ethics Committee (KUERC) under reference number PKU/2790/11915. Further approval was sought from the National Commission for Science, Technology and Innovation (NACOSTI) with license reference number NACOSTI/P/23/30057.

## Results

### Descriptive analysis of delayed and non-delayed PTB cases

The median age of the respondents was 33 years (IQR of 26–43.8 years), with the mean age of 35 years. Most delays were patient-related, with 153(69%) experiencing delays of ≥21 days. In contrast, 69 (31%) sought care within 20 days. Health system delays were reported for 43 (19.4%) patients, while 179 (80.6%) were diagnosed promptly. The mean duration of patient delay was 58.5 days (median: 40; range: 0–516), and for health system delays, it was 17 days (median: 3; range: 0–121).

### Spatial patterns of delayed and non-delayed PTB cases

**Fig 1:**
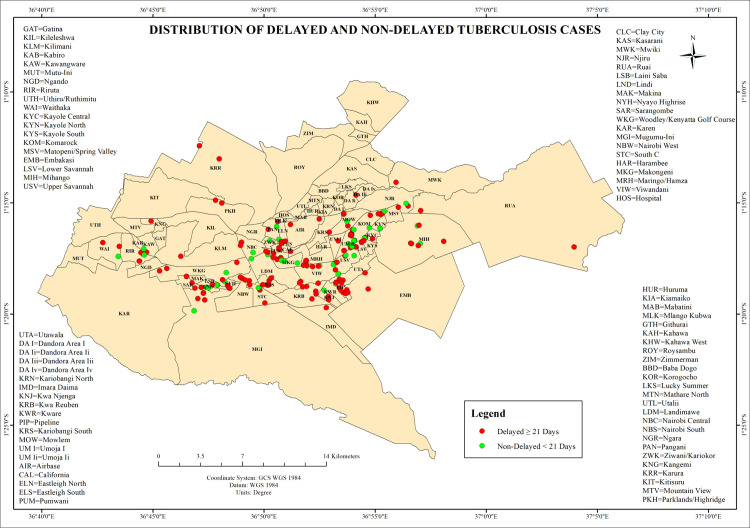
The map illustrates the distribution of delayed (red) and non-delayed (green) tuberculosis (TB) cases across Nairobi County.

Spatial analysis revealed geographic disparities. Delayed cases were concentrated in informal settlements, including Kibera, Mathare, Mukuru kwa Njenga, Mukuru kwa Reuben, Kasarani, Embakasi, Kayole, Dandora, and parts of Dagoretti. Non-delayed TB cases (31%) were predominantly observed in formal, affluent neighborhoods such as the Central Business District (CBD), Westlands, Kilimani, Lavington, Eastleigh, and Lang’ata. These spatial trends are illustrated in Fig 1, which displays delayed cases as red dots and non-delayed cases as green dots overlaid on the ward-level map of Nairobi County (Fig 1).

### Spatial autocorrelation

**Fig 2:**
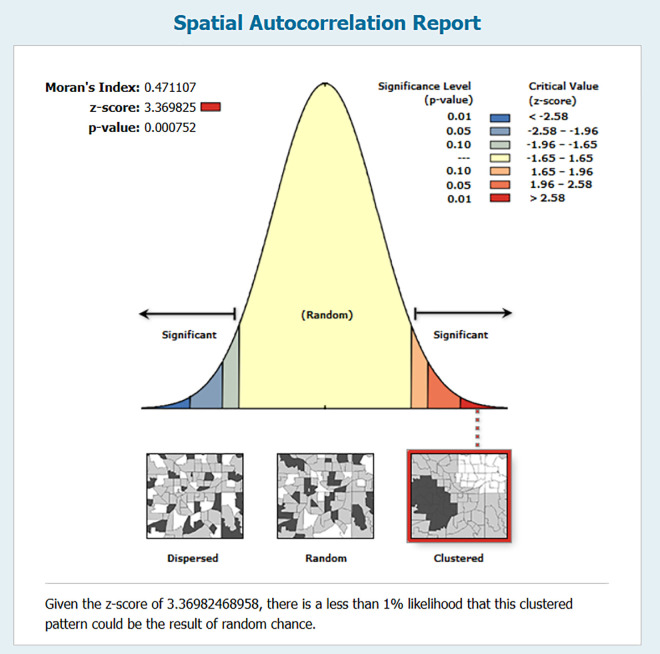
Spatial autocorrelation report.

The calculated Moran’s I value was 0.471, with a corresponding Z-score of 3.370 (Z = ±2.58, 95% confidence) and a p-value < 0.001, which indicated that the observed spatial pattern occurring by random chance was less than 0.001% (Fig 2).

### Hot spot analysis

**Fig 3:**
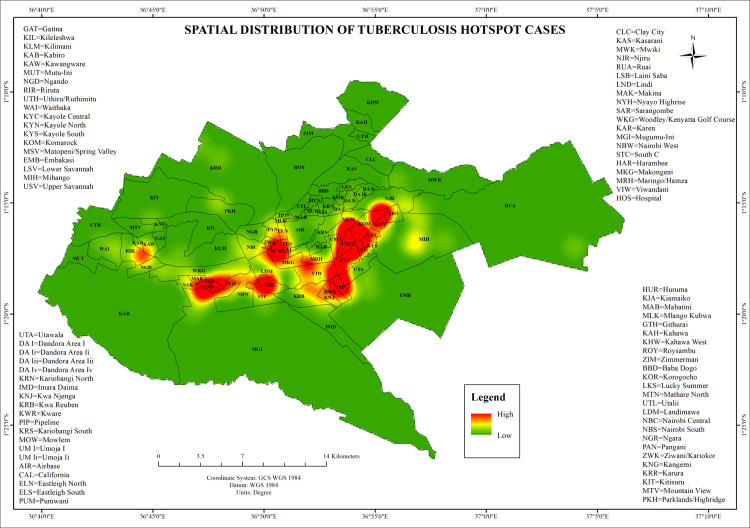
This map illustrates the hot and cold spots of delayed and non-delayed tuberculosis (TB) diagnoses across Nairobi County.

Getis-Ord Gi* identified statistically significant hot spots (z-score >2.58, p < 0.01) in Riruta, Makina, Kayole, Pipeline, Umoja, Eastleigh North, and Komarock. Transitional zones characterized by moderate clustering (z = 1.65–1.96, p < 0.001) were observed in South B, South C, Njiru, Mihango, and Viwandani. Cold spots (z < −2.58, p < 0.001) occurred in Karen, Lang’ata, Runda, and Muthaiga, representing areas with predominantly non-delayed diagnoses. These results are visualized in Fig 3 using intensity gradients and support the identification of high-risk and low-risk zones relevant to programmatic response.

### Local cluster and outlier analysis

**Fig 4:**
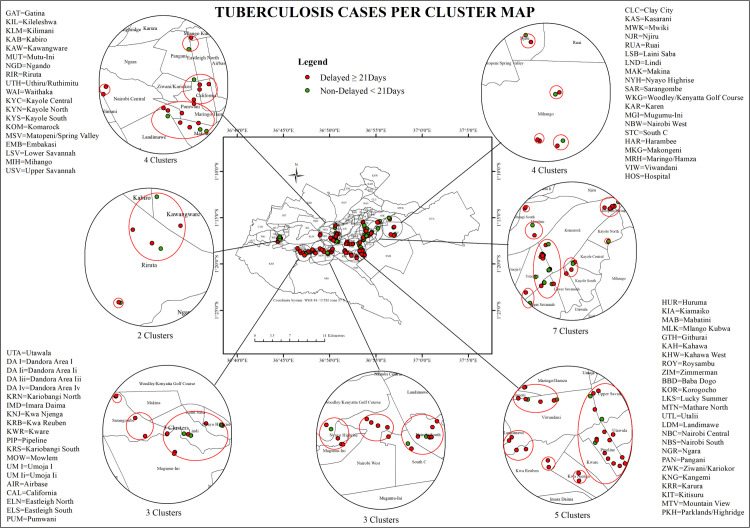
This map illustrates the clustering of delayed and non-delayed (TB) cases in Nairobi County, highlighting clustering patterns of delayed and non-delayed diagnoses.

To further explore spatial clustering, Anselin’s Local Moran’s I was employed to assess local patterns, detect outlier spatial clusters, and identify outliers in TB diagnostic delays. Local Moran’s I identified the extent of spatial association between a feature and its neighboring values, providing a z-score and p-value to determine the statistical significance of clustering or outlier patterns. A z-score greater than ±1.96 indicated clustering at a 95% confidence level, while a p-value below 0.05 confirmed the result was unlikely due to random chance.A total of 28 statistically clusters TB cases were identified through spatial proximity mapping, used to visualize concentrations of delayed and non-delayed diagnoses. Separately, Anselin’s Local Moran’s I analysis was applied to assess local spatial autocorrelation, identifying 73 statistically significant local clusters (*p* < 0.05). To identify localized spatial patterns of diagnostic delay, Anselin’s Local Moran’s I (LISA) was applied. Local clusters were categorized as follows:(1) High-High (HH): Wards with high delay values surrounded by areas with similarly high values, indicating spatial hotspots,(2) Low-Low (LL): Wards with low delay values surrounded by similarly low-delay neighbors, suggesting spatial coldspots.,(3) High-Low (HL): Wards with high delay values surrounded by low-delay neighbors, representing potential spatial outliers, and (4) Low-High (LH): Wards with low delay values surrounded by high-delay neighbors, also considered spatial outliers (Fig 4).

**Fig 5:**
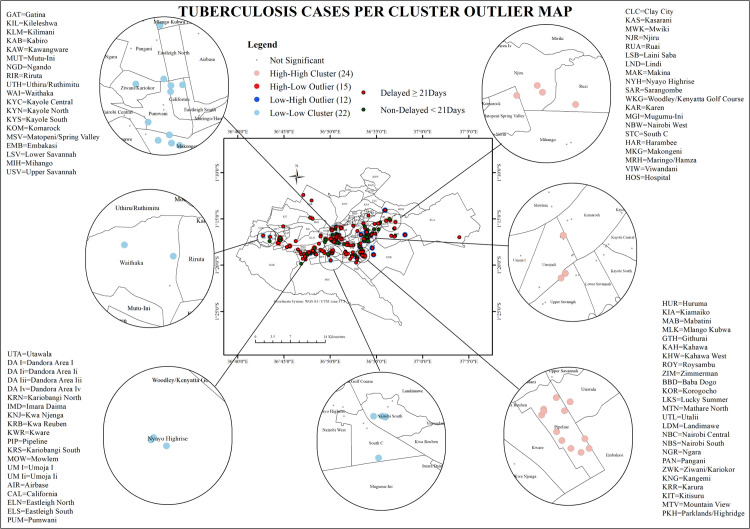
This map illustrates the spatial distribution of tuberculosis (TB) cases in Nairobi County, highlighting clustering patterns of delayed and non-delayed diagnoses.

Among them, 24 were classified as H-H clusters. These H-H clusters were found in Kayole, Njiru, Pipeline, Komarock, Umoja, Embakasi, and Mukuru. 15 H-L12 L-Hand 22 L-L clusters. L-L clusters were identified in Waithaka, Nairobi South, Lang’ata, Nyayo Highrise, and South C (Fig 5).

Spatial outliers with statistical significance evaluated at p < 0.05. A total of 27 wards were classified as outliers, consisting of 15 H-Land and 12 L-H types. H-L outliers, including wards such as Kayole and Umoja, exhibited high TB diagnostic delay values (mean delay > 210 days), yet were surrounded by neighbors with significantly lower delays (mean delay < 100 days). These wards had Local Moran’s I Z-scores close to zero, ranging from 0.23 to 0.65, and p-values indicating statistical significance (p = 0.032). Conversely, L-H outliers, such as Nairobi South and Riruta, were characterized by low diagnostic delays (mean delay < 20 days) while being bordered by wards with substantially higher delays (mean delay > 21 days). These outliers exhibited negative Z-scores between –0.48 and –0.79 and met the statistical threshold of p < 0.05. The local anomalies quantified deviations from the general spatial pattern and illustrated non-uniformity in TB diagnostic delays across adjacent geographic units.

**Fig 6:**
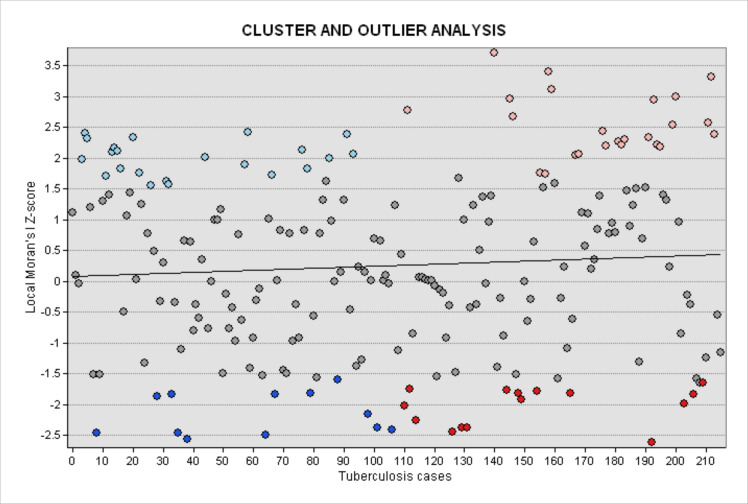
Cluster and Outlier scatterplot of delayed and non-delayed Tuberculosis cases in Nairobi County.

The spatial clustering and outlier patterns were visualized through a scatter plot that mapped Local Moran’s I Z-scores (Y-axis) with TB case counts (X-axis). H-H clusters appeared in the upper-right quadrant of the plot, characterized by Z-scores greater than +2.58 and TB cases exceeding 160 cases. These included Pipeline (Z = 3.21), Kayole (Z = 3.08), and Embakasi (Z = 2.94). L-L clusters were positioned in the lower-left quadrant, with Z-scores below –2.58 and TB case counts below 50, as observed in Lang’ata (Z = –2.81) and Waithaka (Z = –2.67). Spatial outliers were aligned along the horizontal axis, where Z-scores were near zero but showed statistically significant differences from surrounding spatial units. H-L outliers, such as Kayole and Umoja, had Z-scores ranging from 0.23 to 0.65, while L-H outliers, such as Nairobi South and Riruta, had Z-scores between –0.48 and –0.79. All clusters and outliers reported p-values < 0.05, confirming statistical significance at the 95% confidence level (Fig 6).

### Spatial variations of diagnostic health facilities associated with the distribution of Mycobacterium tuberculosis

The spatial distribution of tuberculosis (TB) diagnostic delays across wards in Nairobi County exhibited marked heterogeneity. Using the predefined classification thresholds, several areas demonstrated significant diagnostic delays. The most pronounced delays were observed in the western and northeastern wards, including Kibra and Dandora. Central wards, such as Kilimani and parts of Westlands, demonstrated comparatively shorter delays but still exceeded the threshold for non-delayed classification in peripheral wards. Njiru and Kasarani showed moderate delays, while Embakasi East and Embakasi South had the shortest delays.

### Distance to diagnosing health facilities

**Fig 7:**
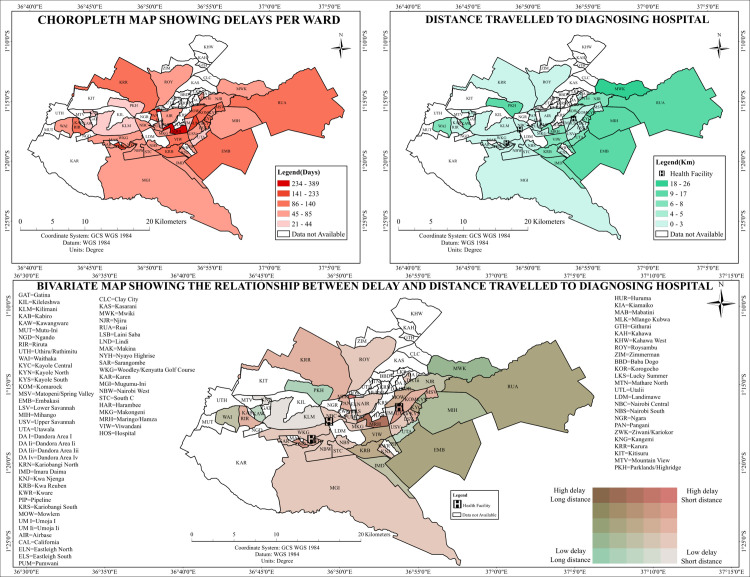
Spatial Analysis of Tuberculosis Diagnostic Delays and Distance to the diagnostic health facility.

Spatial analyses of travel distance and duration to TB diagnostic facilities revealed notable geographic variation across Nairobi County. Central wards such as Starehe, Kamukunji, and Makadara had the shortest mean travel distances to diagnostic facilities. Longer distances were recorded in peripheral wards, including Ruai, Utawala, and parts of Embakasi West (Fig 7).

### Time to diagnosing health facilities

**Fig 8:**
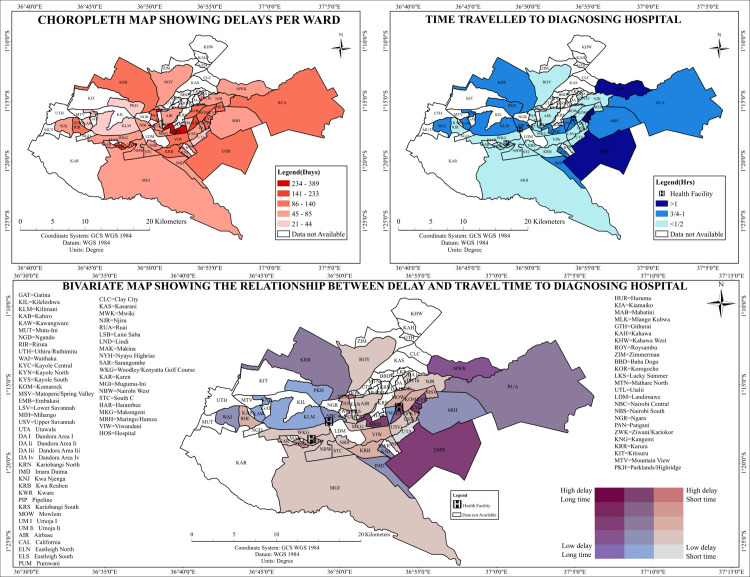
Spatial Analysis of Tuberculosis Diagnostic Delays and Time travel to the diagnostic health facility.

Analysis of travel time to TB diagnostic facilities (Fig 7, Top Right) revealed apparent spatial variation across Nairobi County. Peripheral wards, including Ruai, Kamulu, Mihango, Utawala, and Embakasi West, exhibited the most extended travel durations. Central wards such as Starehe, Kamukunji, and Makadara had the shortest reported travel times. Similarly, Kilimani and Westlands demonstrated reduced travel times due to their proximity to high-density health facility zones (Fig 8).

### Bivariate patterns

#### Relationship between delays, travel distance, and travel time.

The spatial analysis revealed substantial variation in travel time and distance to health facilities across Nairobi County. Figs 7 and 8 (Bottom Panels) depict the integrated spatial distribution of these indicators. Peripheral wards, including Ruai, Utawala, Mihango, and Dagoretti South, were identified as high-delay and long-travel zones. These areas were prominently visualized in dark red and purple, representing spatial zones with compounded geographic and temporal burdens. Central wards, such as Starehe, Kamukunji, and Embakasi East, were mapped in lighter hues, reflecting minimal delays and shorter travel distances or times. Some wards exhibited spatial discordance. Kibra, for example, was classified as a short-distance and short-travel-time area, yet high diagnostic delays were reported. Westlands and Dagoretti North displayed similarly asynchronous spatial patterns, where short travel durations co-occurred with elevated diagnostic delays. These wards appeared in mixed tones on the bivariate maps, indicating non-alignment between proximity to care and diagnostic timeliness. The results delineated spatial zones with consistent or divergent delay-access relationships across the county (Fig 7 and Fig 8).

#### Relationship between diagnostic delays and household income.

**Fig 9:**
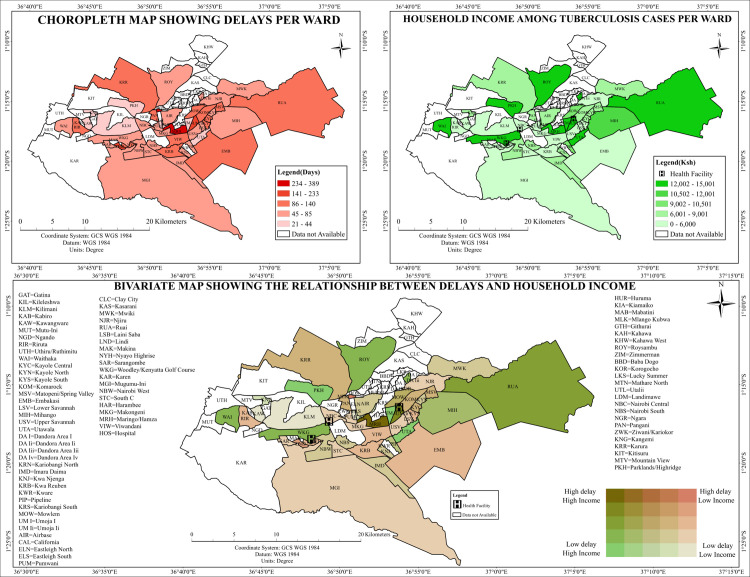
Bivariate map of Tuberculosis Diagnostic Delays and Household income in Nairobi County.

The spatial distribution of diagnostic delays and household income across Nairobi County demonstrated marked heterogeneity at the ward level. Fig 9 (Top Left) revealed prolonged diagnostic delays in the wards Dagoretti North, Dagoretti South, Kibra, and Westlands. Simultaneously, the choropleth map of household income (Fig 9, Top Right) showed that these same wards spanned both high and low-income classifications, with Westlands reporting higher household incomes while Kibra reflected lower-income levels.

The bivariate spatial overlay (Fig 9, Bottom Panel) revealed four distinct categories of delay-income relationships. Wards such as Karen and Lang’ata were mapped in dark green, indicating high household income levels coinciding with low diagnostic delays. In contrast, Kibra, Mathare, and Korogocho appeared in dark brown, representing wards with both low-income and high diagnostic delays. These areas exhibited spatial alignment between socioeconomic vulnerability and delayed access to TB diagnosis. Interestingly, spatial mismatches were also observed. Westlands and Dagoretti North were mapped in light brown, reflecting high-income levels but elevated diagnostic delays. Conversely, areas such as Ruaraka and parts of Embakasi South were colored in light green, showing relatively low household incomes alongside minimal diagnostic delays (Fig 9).

### Multivariate spatial analysis of tuberculosis diagnostic delays, travel distance, travel time, and household income in Nairobi County, Kenya

**Fig 10:**
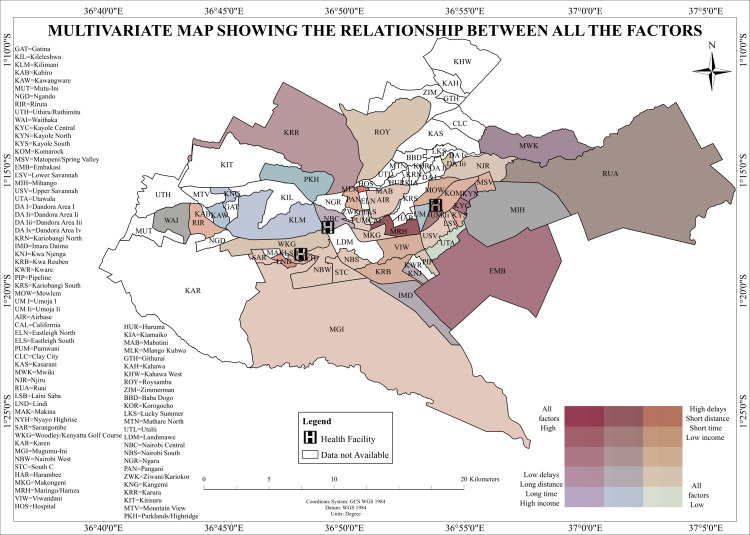
Multivariate map of tuberculosis diagnostic delays, travel distance, travel time, and household income in Nairobi County.

This study conducted a multivariate spatial analysis to integrate diagnostic delays, travel distance, travel time, and household income within a composite mapping framework. The spatial intersection of these four variables illustrated distinct intra-urban diagnostic risk profiles. Kibra, Mathare, and Korogocho were identified as high-delay, short-distance, low-income areas. Despite their geographic proximity to healthcare facilities and relatively minimal travel time, these areas, visualized in dark red on the multivariate relationship map, exhibited substantial diagnostic delays. This spatial configuration indicates overlapping structural and socioeconomic constraints affecting healthcare access. Westlands, Karen, and Dagoretti North were classified as high-delay, long-distance, high-income areas. These wards, mapped in light brown, experienced prolonged diagnostic intervals despite advantageous socioeconomic conditions, suggesting the influence of non-geographic factors overlaid on broader spatial separation. Ruai and Utawala represented low-delay, long-distance, low-income zones. Depicted in light green, these peripheral wards achieved timely TB diagnoses despite geographic and economic disadvantages, highlighting spatial resilience under constrained conditions. Lang’ata, Runda, and adjacent wards were identified as low-delay, short-distance, high-income zones. These areas, mapped in dark green, exemplified optimal spatial and socioeconomic conditions for timely TB diagnosis. Fig 9 presents the multivariate relationship map, providing a detailed spatial synthesis of diagnostic delay patterns shaped by the interplay of geographic accessibility and socioeconomic context. The multivariate relationship map offers a spatial synthesis of multiple delay-influencing factors across the study area (Fig 10).

### Spatial accessibility and geographic coverage of health facilities

#### Service area analysis of health facilities.

The service area analysis applied 2 km, 5 km, and 8 km Euclidean buffers around existing public health facilities to evaluate geographic accessibility to tuberculosis (TB) diagnostic services in Nairobi County. Central wards such as Starehe, Kamukunji, and Makadara were fully covered within the 2 km buffer zone, indicating high spatial accessibility. These areas corresponded to wards with a higher density of public health infrastructure.

**Fig 11:**
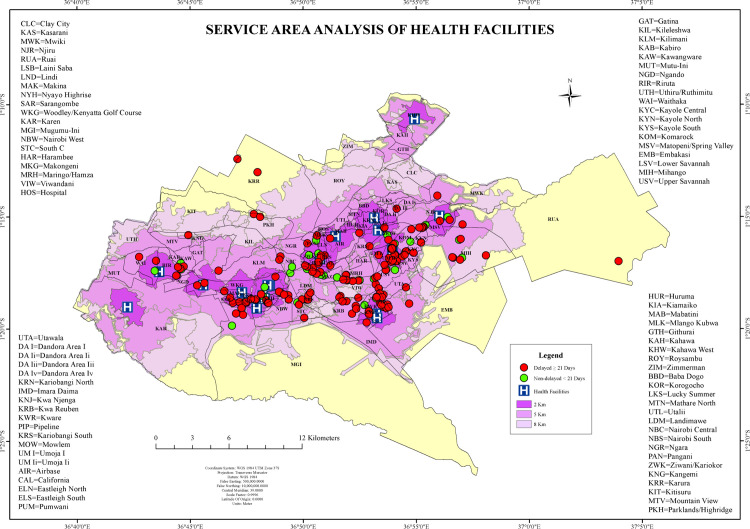
service area analysis of public health facilities in Nairobi County, Kenya.

In contrast, peripheral wards, including Ruai, Utawala, Mihango, and Karen, were either partially or entirely excluded from the 8 km service coverage. Across the county, 65.4% of delayed TB cases were located outside the 5 km buffer zone, and these wards showed a mean distance of 3.2 km from the nearest facility and a mean diagnostic delay of 184 days. In comparison, wards within the 2 km buffer zone exhibited significantly shorter delays and proximity, with most non-delayed TB cases clustering within these centrally located catchment areas (Fig 11).

#### Location-allocation of new health facilities.

**Fig 12:**
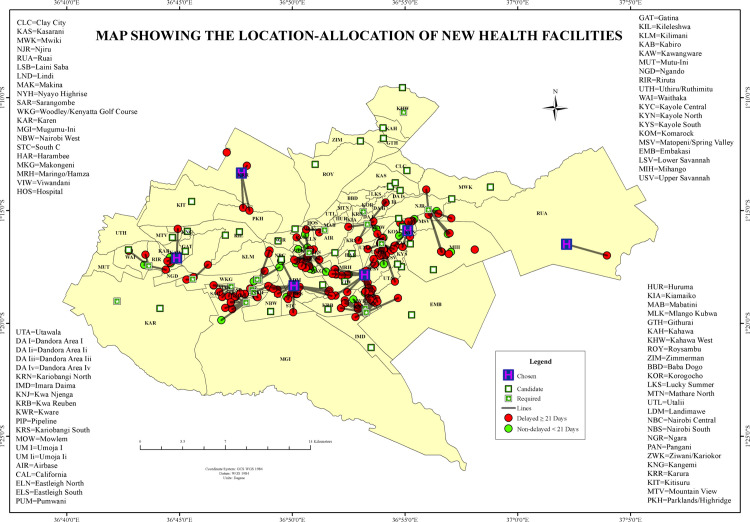
Location-allocation of new health facilities in Nairobi County, Kenya.

The location-allocation analysis identified candidates and optimal locations for new health facility placement, considering the spatial distribution of TB case burden and underserved populations (Fig 12). Existing facilities were concentrated in central areas, notably in Starehe, Kamukunji, and Makadara, while the peripheral wards exhibited marked facility gaps. Candidate sites (n = 12) were proposed across underserved zones, and six optimal sites were selected based on spatial demand and burden alignment. The selected locations, primarily within Kibra, Mathare, and Korogocho, coincided with areas reporting 72.1% of delayed TB cases and a mean diagnostic delay of 202 days. The average network distance from these delayed cases to the nearest current facility was 4.5 km, exceeding the mean distance for non-delayed cases in central zones (Fig 12).

#### Closest facility route network analysis.

**Fig 13:**
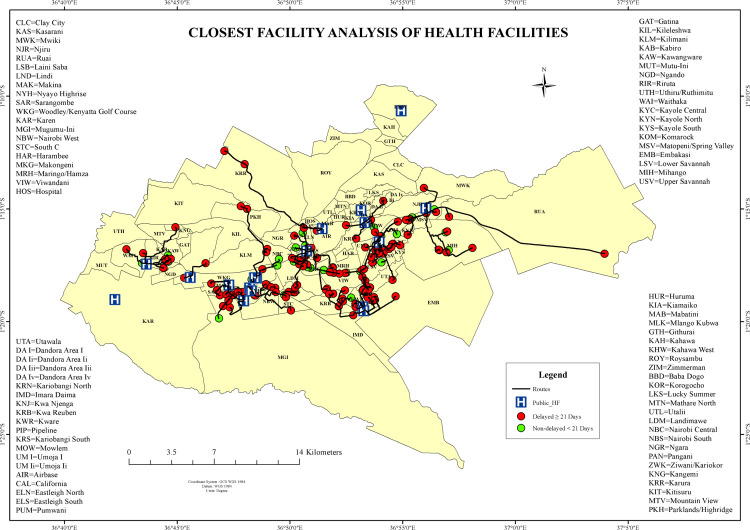
Closest Facility Route Network Analysis showing distance to the diagnosing health facilities in Nairobi County, Kenya.

Network-based route analysis determined the shortest travel path from the patient’s residence to the closest public TB diagnostic facility (Fig 13). Results revealed that delayed TB cases were primarily concentrated in peri-urban and peripheral wards such as Ruai, Utawala, and Mihango, where the mean travel route distance was 4.9 km, and the mean diagnostic delay reached 196 days. These wards recorded maximum route distances of up to 18 km. Conversely, non-delayed TB cases were predominantly found in wards such as Lang’ata, Nairobi Central, and Westlands. These areas exhibited average route distances of less than 2 km, with rapid diagnostic timelines corresponding to more accessible health services. The spatial network analysis quantitatively reinforced disparities in proximity between delayed and non-delayed TB diagnoses across Nairobi County.

## Discussion

This study provides a novel geospatial analysis of the distribution of delayed and non-delayed TB cases in Nairobi County using individual-level geolocation data. By moving beyond traditional area-based surveillance datasets, we employed patient-specific geocodes integrated with healthcare accessibility and socioeconomic parameters to access spatial clustering patterns associated with diagnostic delays. To our knowledge, this is the first study to use individual-level geocodes to analyze delays and non-delays in PTB diagnosis. This study integrated health facility accessibility and socioeconomic factors instead of relying on surveillance data based on geographic units. This approach was useful for spatial precision. Socioeconomic conditions, travel time, and geographic proximity to health facilities were overlaid on diagnostic delay data to enable integrated spatial analysis of access-related determinants.. Our findings indicated substantial disparities consistent with global evidence, particularly within urban contexts in sub-Saharan Africa [6,8].

Significant clustering (Moran’s I = 0.471, p < 0.001) and hot spot analyses showed delays concentrated in informal settlements. These zones, generally characterized by low socioeconomic status, high population density, and reduced healthcare infrastructure, which may lead to diagnostic delays. These findings reflected papers published from Ethiopia and South China that also identified TB clusters where populations were outward bound and were situated in areas of socioeconomic deprivation [9,10]. Cold spots, reflecting lower rates of diagnostic delay, which correspond with the study conducted in South Korea that stated cold spots were situated in areas with significant population growth, the best urban infrastructure, and an affluent socio-economic status [11]. Conversely, a study in Uganda did not observe strong spatial clustering of diagnostic delays [12]. Individual-level and health system factors were more influential than our Nairobi findings. This difference may have come from variations in study design and methodological consequences. Our use of individual-level geocoded data and network-based route and service area analyses enabled a finer-scale detection of spatial disparities that aggregate data might overlook.

The findings of the study are consistent with the reports from Ethiopia, Uganda, and other regions in sub-Saharan Africa, which highlight the role of patient and health system factors in contributing to delayed TB diagnoses [13,14]. Our study found that patient-related delays had a median of 40 days, whereas health system delays were comparatively low, with a median of 17 days. Other studies have reported that the median delay from the onset of symptoms to the initial healthcare visit ranges from 35 days to 38 days in one setting in Iran. Additionally, the diagnosis of pulmonary TB in patients showed delays exceeding 30 days [15–17]. Despite the comparatively shorter health system delays observed in our study, these delays reveal persistent barriers to timely diagnosis and care. This observation aligns with findings from Ethiopia, where delays at the health system level were attributed to health facility accessibility [18,19]. The persistent factors continue to pose constraints to timely TB diagnosis in informal settlements in Nairobi.

To further explore the spatial distribution of the diagnostic delays, the study applied Anselin Local Moran’s I analysis across Nairobi County, identifying 73 statistically significant spatial clusters.. This spatial polarization reflects clear disparities in TB diagnostic timeliness. High-High clusters are primarily located in peri-urban and densely populated informal settlements. The clusters are defined as localized areas in which delays in diagnosis are accompanied by other adjacent wards that are dealing with similarly problematic delays. As such, these clusters likely represent a shared structural vulnerability across wards, similar to access to health services and/or lower socioeconomic status. This is consistent with the results of the study conducted in China, where 4 aggregation areas were identified with H-L regions, indicating that cases were more prevalent and less prevalent in the surrounding areas. In contrast, L-L clusters highlight regions with non-delayed diagnoses, likely supported by favorable health system indicators and socioeconomic conditions. These areas may benefit from increased health-seeking behavior.

This pattern aligns with the findings from Kashgar, China, where the application of a local spatial autocorrelation model identified H-L and L-H TB clusters [9]. The proximity of these clusters to well-developed areas with good access to health services could positively affect the neighborhoods. Similarly, in Nairobi, H-L outliers, where low-delay neighbors surround wards with high delays, may indicate areas of non-delay diagnosis [9]. Moreover, the relationship of health facility distribution with TB case detection in Kashgar indicated that health areas with higher densities of health facilities had higher TB case detection. High-Low healthcare locations may have fewer diagnostic delays, while low-resource health facilities would carry the burden of amplified diagnostic delays. Areas with robust diagnostic services may detect cases earlier, while under-resourced regions face compounded delays. The movement of populations from underserved areas to better-equipped neighborhoods, especially for diagnosis, could further amplify these disparities, contributing to urban TB transmission networks. Thus, the Nairobi study echoes global findings that highlight the importance of spatially targeted TB control strategies. Addressing the needs of H-H clusters and outlier regions requires expanding coverage of diagnostic services and ensuring that existing health services are effectively utilized and accessible to all through outreach and mobile services.

In this study, we assessed health facility accessibility of TB diagnostic timeliness in Nairobi County. The diagnostic delays were significantly associated with peripheral wards such as Ruai, Mihango, Utawala, and Dagoretti South. These areas exhibited longer travel distances, with a mean of 4.9 km, which were measured using Euclidean distances, and prolonged diagnostic delays (up to 196 days), underscoring the spatial inequities in diagnostic service distribution. The findings are consistent with research findings from Lima, Peru, that estimated pedestrian travel time and reported stronger predictors of community-based TB screening uptake [12,20]. This reinforces our observation that there are clusters of delayed diagnosis even in wards proximate to health facilities. Instead, individual-level factors like health-seeking behaviour play a key role in mediating access to diagnostic services [21]. This underscores the need to address both spatial and behavioural factors when designing TB control interventions in resource-limited urban settings.

A study conducted in Ethiopia examined access to primary healthcare services in rural communities and found that proximity to health facilities significantly influenced healthcare-seeking behavior, leading to delays in diagnosis [22]. Our results echoed this finding, revealing that 65.4% of delayed tuberculosis (TB) cases were located outside the 5 km service area buffer. Additionally, even in some wards with nearby facilities, delays continued to occur, likely due to the patients’ healthcare-seeking behaviour.

The location-allocation analysis in our study identified underserved hotspots where placing mobile TB screening units or additional health facilities would maximize coverage for high-delay clusters. Similar findings from South Africa showed that reducing TB burden requires making services not just available, but also physically accessible to the people who need them [23]. Similar to our findings on the delayed diagnosis of TB, a study in Delaware showed that access to mammography services varies with how and where resources are placed, highlighting the shared need for better spatial planning to reduce health disparities [24]. Our study found a notable finding that the presence of spatial mismatches, such as in Kibra, where high delays existed despite proximity to health facilities. These “high-delay, short-distance” zones suggest functional inaccessibility, likely driven by health-seeking behavior and economic constraints, or facility inefficiencies. Similar mismatches were reported in the Ethiopian and South African contexts, where geographic proximity did not equate to access, especially in underserved or overcrowded urban areas [25,26]. These findings confirmed that TB control efforts in urban settings must move beyond geographic proximity alone and address access. Integrating geospatial analyses such as service area analysis and location-allocation models into routine TB program design could significantly improve targeting of high clusters of delay zones and optimize resource allocation.

Our study showed that geographic accessibility was significantly associated with diagnostic delays. Peripheral wards such as Ruai, Utawala, and Mihango experienced longer travel distances and durations to health facilities, correlating strongly with delayed diagnoses. This aligns with prior research emphasizing distance as a barrier to timely healthcare access and TB diagnosis [27]. Our study used location-allocation analyses to identify 42 potential locations for diagnostic service points. This analysis revealed significant infrastructure gaps, particularly in informal settlements and peripheral areas. These findings support global health strategies that support decentralized, community-based healthcare systems aimed at improving diagnostic timeliness. [28,29].

The bivariate and multivariate analyses revealed a significant association between diagnostic delays and socioeconomic factors in low-income wards like Kibra, Mathare, and Korogocho. These findings strengthen evidence linking poverty, overcrowding, and increased TB incidence in informal settlements [30,31]. Interestingly, spatial divergences were noted in areas such as Westlands and Dagoretti North, where higher incomes did not necessarily correlate with timely diagnoses, indicating other influencing factors such as health-seeking behaviour [32–34].

### Public health implications.

These findings demonstrate significant public health implications for tuberculosis control in urban low-resource settings. Delays were notably concentrated in informal settlements and peripheral wards, underscoring the need for spatially targeted interventions, including mobile diagnostic units and screening services. Additionally, the association between diagnostic delays, longer travel distances, and lower household income emphasizes the importance of integrating spatial equity and socioeconomic vulnerability into TB program planning. In wards proximate to diagnostic health facilities, prolonged delays suggest underlying health-seeking behavior and systemic barriers, warranting community awareness strategies. Integrating patient-level geospatial data and spatial analytic tools into routine surveillance could enhance the precision of resource allocation, supporting national and global efforts to reduce TB morbidity and mortality in urban, resource-constrained settings.

### Strengths and limitations

The primary strength of this study is the use of individual-level geocoded data combined with bivariate and multivariate spatial analysis. The approach allowed precise identification of TB diagnostic delay patterns in Nairobi county. Previous studies relied on aggregated surveillance data. This method provided detailed insights into spatial diagnostic delays across wards with varying socioeconomic backgrounds. Our study was implemented using limited personal resources, which constrained the sample size and coverage and did not consider temporal dynamics or established causal pathways between accessibility, socioeconomic factors, and tuberculosis diagnostic delays; it only observed associations measured at a single point in time.

## Conclusion

The findings of this study revealed strong clustering of delayed tuberculosis diagnoses in both informal settlements and peri-urban wards of Nairobi County. The identified informal settlements were marked by significant diagnostic delays coupled with limited geographic access to health facilities and compounded by socioeconomic vulnerabilities. Additionally, peripheral wards exhibited longer travel distances and travel times to TB diagnostic centers, which were strongly associated with prolonged diagnostic delays. We recommend the integration of mobile TB clinics and strengthening of community-based outreach services, particularly in high-delay clusters, identified through spatial analyses by the Ministry of Health (MOH) and the county health department. This strategy should be supported by location allocation analyses to optimize geographic coverage and reduce travel burdens. Future research should prioritise the longitudinal designs to elucidate causal pathways between geographic accessibility, socioeconomic factors, health-seeking behavior, and tuberculosis diagnostic delays, thereby addressing the temporal limitations inherent in the current cross-sectional approach. Future research should include larger, population-representative samples for more robust spatial inference to improve the generalizability of findings
